# Functional significance of cuckoo *Cuculus canorus* calls: responses of conspecifics, hosts and non-hosts

**DOI:** 10.7717/peerj.5302

**Published:** 2018-09-18

**Authors:** Piotr Tryjanowski, Federico Morelli, Tomasz S. Osiejuk, Anders Pape Møller

**Affiliations:** 1 Institute of Zoology, Poznań University of Life Sciences, Poznań, Poland; 2 Faculty of Environmental Sciences, Department of Applied Geoinformatics and Spatial Planning, Czech University of Life Science, Prague, Czech Republic; 3 Department of Behavioural Ecology, Institute of Environmental Biology, Faculty of Biology, Adam Mickiewicz University of Poznań, Poznań, Poland; 4 Department Ecologie Systématique Evolution, CNRS, AgroParisTech, Université Paris Sud (Paris XI), Orsay Cedex, France

**Keywords:** Anti-parasite behaviour, *Cuculus canorus*, Cuckoo, Mobbing

## Abstract

Male cuckoos *Cuculus canorus* produce calls that differ in number of syllables depending on environmental conditions and presence of male and female conspecifics. Why different males produce so repeatable calls that vary greatly in duration among males remains an open question. We used playback of cuckoo calls with few or many syllables (hereafter short and long calls), and woodpigeon calls (a control that also produces few or many syllables), predicting that playback of longer cuckoo calls should attract more male cuckoos (if males with such calls are dominant and successfully out-compete other males due to intraspecific competition), and attract more hosts mobbing male cuckoos (cuckoos with such calls and their females attract more hosts because of an increased risk of parasitism). Because cuckoos differentially parasitize hosts away from human habitation, we also tested whether the number of syllables in cuckoo calls differed with distance from buildings. Playback showed significant effects of number of syllables in cuckoo calls, but not woodpigeon *Columba palumbus* calls, with an additional effect of distance from human habitation decreasing the response to playback. These findings are consistent with the hypothesis that longer cuckoo calls, especially played back near human habitation, attract more conspecifics and hosts than shorter calls. To the best of knowledge this is the first study showing that cuckoo call response modified both other cuckoo individuals, as well as hosts response.

## Introduction

A general feature of animal sexual communication is the exaggeration of signals with longer, larger or stronger signals being transmitted across longer distances and having stronger impact on receivers (Ryan & Keddy-Hector, 1992). Thus, signals with more repeats are generally more efficient and attractive than those with fewer repeats (Ryan & Keddy-Hector, 1992; Catchpole & Slater, 2008; Searcy & Nowicki, 2005). Generally, this applies to vocal, visual, chemical and other signals (Bradbury & Vehrencamp, 2011).

Male common cuckoos *Cuculus canorus* produce calls that differ in the number of ‘cu-coo’ syllables depending on quality of habitat and presence of male and female conspecifics (Møller, Liang & Díaz, 2016). Why males produce so repeatable calls that vary strongly in number of repeat ‘cu-coo’s among individuals remains an open question. Cuckoos generally avoid human habitation, especially urban areas, and hosts benefit from breeding close to houses by reducing parasitism rates (Møller et al., 2016). However, cuckoos in villages may parasite local hosts with high intensity (Antonov et al., 2007). Moreover, cuckoos flew away at much longer distances than hosts when approached by a human (Møller, Liang & Díaz, 2016).

Song duration is generally a reliable measure of male quality in many bird species, especially passerines (Gil & Gahr, 2002). In the cuckoo call duration is under strong sexual selection (e.g., Fuisz & De Kort, 2007; Møller, Morelli & Tryjanowski, 2017). However, calling cuckoos are also mobbed by other bird species, including hosts and non-hosts, and the strength of response may depend on cuckoo calls (e.g., Welbergen & Davies, 2009; Trnka & Prokop, 2012; Kleindorfer et al., 2013; Moskat et al., in press). The response of hosts to cuckoos is modulated by reproductive status of hosts and their learning abilities, and it varies among host species (Welbergen & Davies, 2009; Welbergen & Davies, 2011; Campobello & Sealy, 2011). However, detection of a male cuckoo by other birds is costly because it elicits mobbing, and therefore, mobbing may reduce the duration of cuckoo calls (Curio, Ernst & Vieth, 1978; Krama et al., 2012). Thus, we predicted that only males in prime condition produce long calls consisting of many syllables. Nevertheless, studies of the function of the duration of cuckoo calls have mainly been correlative (Wei et al., 2015; Møller et al., 2016; Zsebők, Moskát & Bán, 2017). Generally, non-passerine vocalizations are not based on learning (Ryan & Keddy-Hector, 1992). Calls in general are preferred when containing more repeat syllables. However, experimental tests for such a preference are few (Catchpole & Slater, 2008, but see Khan & Qureshi, 2017).

Here, we experimentally tested the functional significance of the duration of cuckoo calls in playback experiments and measured how cuckoo males responded to playback of cuckoo calls differing in the number of syllables. Furthermore, we tested whether birds mobbed a stuffed cuckoo more strongly when we played back a large rather than a small number of syllables.

We predicted that (1) playback of the male ‘cu-coo’ call would attract more male cuckoos, (2) more heterospecific birds would mob cuckoos when we played back cuckoo calls with more syllables, (3) response to playback would be stronger when cuckoo parasitism of a bird species was more common, and (4) response would be stronger in colonial than in solitarily breeding birds that benefit from multiple conspecifics on the outlook for parasitic cuckoos.

## Methods

### Study area

The study was conducted during May–July 2016 in Wielkopolska province, Poland (52°N, 16°E). Habitats cover mainly mixed farmland, with small patches of forest and waterbodies. Information on the density of cuckoos and potential hosts is reported in Tryjanowski & Morelli (2015). Data were collected at 97 sites distributed over agricultural landscapes with minimum distances of 1 km between study sites.

### Playback stimuli preparation

To avoid similarity in call structure between chosen calls, we used synthetic calls of cuckoos and woodpigeons (*Columba palumbus*), similar to the system described by Campobello & Sealy (2011). As model calls, we chose high quality samples of local territorial calls of common cuckoo and common woodpigeon as controls. Based on these natural calls we prepared their synthesized versions with Avisoft SASLab Pro 5.2 (Specht, 2016). The synthetic calls were based on their natural equivalent in the following steps: (1) creation of sonogram, (2) scanning of frequency contour and amplitude envelope, and (3) saving as WAV file. For scanning we used automatic three-threshold element separation and appropriate threshold relative to the maximum signal amplitude. Synthetic calls were similar acoustically and they looked very similar on sonograms in comparison with natural samples.

Synthetic calls were passed into WAV files prepared for broadcasting during experiments. To avoid pseudo-replication, synthetic calls, both cuckoo and woodpigeon, were prepared using records of many individual birds. Broadcast sounds begin with 60 s of silence, which allows the observer to set up the loudspeaker and recede at a standard distance from the loudspeaker before the start of playback. After 60 s the loudspeaker, located under small wooden boards to which was attached a stuffed male cuckoo (randomly chosen among four different individuals), reproduced synthetic calls with the following pattern:

(a) Treatment cuckoo—short duration: (5 cuckoo calls within 6 s plus 54 s of silence) repeated 5 times;
(b) Treatment cuckoo—long duration: (25 cuckoo calls within 35 s plus 25 s of silence) repeated 5 times;
(c) Control woodpigeon—short duration: (2 woodpigeon calls within 6 s plus 54 s of silence) repeated 5 times;
(d) Control woodpigeon—long duration: (9 calls within 35 s plus 25 s of silence) repeated 5 times.

As the duration of cuckoo and woodpigeon calls are different we used a different number of calls to keep sound broadcast time to silence time ratio at the same level for low and high intensity experiments, respectively.

### Playback experiments

Calls were broadcast with a waterproofed Creative MUVO mini^®^ loudspeaker and amplitude was standardized for all playbacks.

The order of presentation of the four treatments was randomized, and to avoid habituation only one call was presented daily, and thus on four different days ensuring that all treatments were presented at each site. We took special care to identify whether attracted cuckoos were males (male call) or females (female call, rufous plumage). Technically speaking, we showed that synthetic sound samples resembled natural calls and preliminary playback in the field revealed that different passerines responded to them.

### Field observations

Data were collected 1 May–15 June 2016 6:00–9:30) on days without rain or strong wind. In experimental sites during each of the four trials birds were counted using the point-count method during a period of 5 minutes before start of the playback (I.P.A. method following Järvinen, 1978). Additionally, data from point-counts were summarized and used to describe the breeding bird community around experimental sites where a stuffed cuckoo was presented.

At each of the 97 study sites data were collected four times (treatments a–d, see above), and calls of the cuckoo (short and long) and the woodpigeon (short and long) were presented randomly. In one day only one treatment was presented, and the study site was visited the following days to collect data on reaction to all prepared calls.

In order to avoid errors in recording interactions between cuckoos and other species of birds (mobbing with physical attack, nervous behaviour, changes in song pattern) the response of birds directly to the model cuckoo was included. We paid special attention to distinguish between reacting individuals, which was not difficult because in the majority of cases only one individual per species was detected. Similarly, during field observations of real cuckoos, only call reactions directed towards the cuckoo were used for analyses.

### Coloniality and parasitism rate

We hypothesized that colonially breeding species of birds would react earlier and more strongly to playback and presentation of model cuckoos. Species were classified dichotomously as solitary or colonial according to information in Cramp, Simmons & Perrins (1982-1994).

We predicted that host species with higher parasitism rate had evolved stronger responses to cuckoos due to their more frequent interactions. Hosts of cuckoos were ranked according to how common parasitized species were recorded in Poland (Wesołowski & Mokwa, 2013).

The responses of birds to the model cuckoo were recorded when the birds (1) physically attacked a stuffed bird (mobbing), or (2) approached the dummy and behaved nervously (Moksnes et al., 1991; Røskaft et al., 2002). These two responses were added as the total number of reacting birds of each species.

### Statistical analyses

We used Generalized Linear Models (GLMs) to analyze the reaction of birds after playback (synthetic calls). Models were fitted assuming a Poisson distribution (for number of birds reacting and number of real cuckoo males reacting) after having explored the distribution of variables as suggested by Box & Cox (1964), using the package ‘MASS’ (Venables & Ripley, 2002), and ‘glmmADMB’ in R (Fournier et al., 2012; Skaug, Fournier & Nielsen, 2013). The treatment and the covariates entered in the full model were respectively type of synthetic call (a, b, c, d), date, time and distance to houses.

Model selection was performed using the package ‘AICcmodavg’ in R (Mazerolle, 2016). The best model was selected considering both the smallest Akaike’s Information Criterion (AIC) and largest Akaike weights, because this model has the strongest support for the data (Burnham & Anderson, 2002; Mazerolle, 2016). The confidence intervals for the significant variables selected in the best model were calculated by the Wald method from the package ‘MASS’ (Venables & Ripley, 2002).

Comparison of the number of individuals of host and non-host species reacting was made using *t*-test (Sokal & Rohlf, 1995). All statistical tests were performed with R software (Team, 2016).

### Ethical approval

This study has observational character, and due to Polish national law for this type of study formal consent is not required. Our research did not require approval by the Local Ethical Commission because of the playback experiments do not fall within its authority in Poland according to The Act on Experiments on Animals (Disposition no. 289 from 2005). The playback was kept as short as possible to collect data for the purposes of the current study and we are not aware of any consequences for the subject’s breeding or welfare.

**Table 1:** Bird species recorded in experimental trials mobbing cuckoos with information on number of records, host (1 –host for cuckoo in Poland according to Wesołowski & Mokwa (2013); 0 –non-host) and coloniality (1, colonial species; 0, non-colonial species).

**Species**	**Host**	**Coloniality**	**No. reactions**
*Acanthis cannabina*	0	1	4
*Acrocephalus arundinaceus*	1	0	1
*Acrocephalus palustris*	1	0	22
*Acrocephalus schoebaneus*	1	0	2
*Acrocephalus scirpaceus*	1	0	3
*Alauda arvensis*	1	0	3
*Anthus campestris*	1	0	1
*Anthus pratensis*	1	0	2
*Anthus trivalis*	1	0	1
*Carduelis carduelis*	0	0	3
*Chloris chloris*	0	1	4
*Coccothraustes coccothraustes*	0	0	4
*Corvus corax*	0	0	3
*Corvus cornix*	0	0	1
*Cyanistes cyaneus*	0	0	23
*Delichon urbica*	0	1	16
*Dendrocopos major*	1	0	6
*Emberiza citrinella*	1	0	12
*Emberiza schoeniclus*	1	0	3
*Erithacus rubecula*	1	0	12
*Fringilla coelebs*	0	0	3
*Galerida cristata*	0	0	3
*Garrulus glandarius*	0	0	11
*Hippolais icterina*	1	0	8
*Hirundo rustica*	1	1	143
*Lanius collurio*	1	0	31
*Lanius excubitor*	0	0	14
*Lululla arborea*	1	0	3
*Luscinia meharhynchos*	0	0	3
*Miliaria calandra*	0	0	2
*Motacilla alba*	1	0	33
*Motacilla flava*	1	0	22
*Oriolus oriolus*	0	0	3
*Passer domesticus*	0	1	4
*Passer montanus*	0	1	24
*Parus ater*	0	0	1
*Parus major*	1	0	38
*Parus montanus*	0	0	3
*Phoenicurus ochruros*	1	0	5
*Phoenicurus pheoenicuroides*	1	0	3
*Phylloscopus collybita*	1	0	14
*Phylloscopus sibilatrix*	1	0	2
*Phylloscopus trochilus*	1	0	11
*Pica pica*	0	0	9
*Riparia riparia*	1	1	2
*Saxicola rubetra*	1	0	3
*Saxicola torquata*	1	0	1
*Sitta europea*	0	0	1
*Sturnus vulgaris*	1	1	26
*Sylvia atricapilla*	1	0	10
*Sylvia communis*	1	0	8
*Sylvia curruca*	1	0	8
*Sylvia nisoria*	1	0	10
*Troglodytes troglodytes*	1	0	5
*Turdus merula*	0	0	15
*Turdus philomelos*	1	0	3
*Turdus pilaris*	0	1	11
*Turdus viscivorus*	0	0	1

## Results

During 388 experimental trials produced in 97 sites we recorded in total 58 bird species, 34 host and 24 non-host species (Table 1), in total 4,743 individuals. The maximum number of species recorded simultaneously per site was 16 and the maximum number of individuals 84.

**Figure 1:**
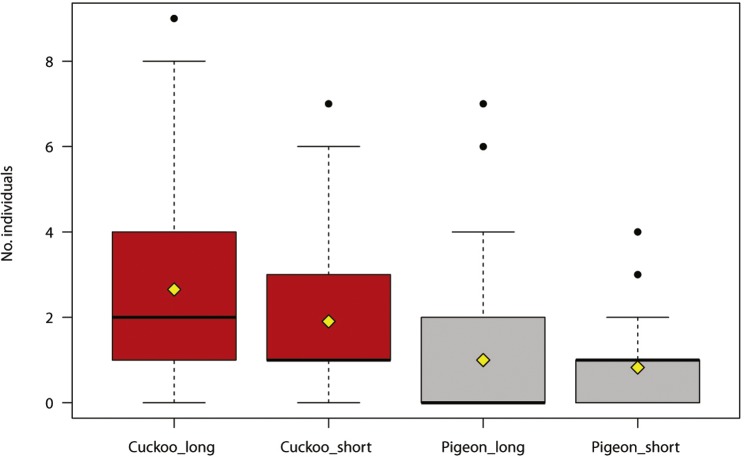
Number of individual birds reacting during experimental play-back of synthetic calls (common cuckoo call in red, wood pigeon call in grey).

The maximum number of reactions recorded per site was 144 (min = 1, SD = 19.78). The number of bird individuals responding to the synthetic call was positively associated with the duration of the cuckoo call, and negatively with the distance to houses (Table 2, Fig. 1). The number of cuckoo males responding to the synthetic call was positively associated with the duration of cuckoo calls (Table 3).

**Table 2:** Results of best model, accounting for variation in the number of bird species reacting to playback, in relation to type of synthetic calls (a, b, c, d), date, time and distance to houses modelled as fixed effects. Significant variables are shown in bold.

**Fixed effects**	**Estimate**	**SE**	***z***	***P***
(Intercept)	−0.156	0.130	−1.199	0.231
Long pigeon call	0.064	0.169	0.378	0.705
**Short cuckoo call**	**0.870**	**0.145**	**5.980**	**<0.001**
**Long cuckoo call**	**1.131**	**0.140**	**8.072**	**<0.001**
**Distance to houses**	**−0.001**	**0.001**	**−4.101**	**<0.001**

**Table 3:** Results of best model accounting for variation in number of real cuckoo males reacting to playback, in relation to type of synthetic calls (a, b, c, d), date, time and distance to houses modelled as fixed effects. Significant variables are shown in bold.

**Fixed effects**	**Estimate**	**SE**	***z***	***P***
**(Intercept)**	**−2.378**	**0.337**	**−7.054**	**<0.001**
Long pigeon call	0.149	0.035	−0.043	0.966
**Short cuckoo call**	**2.820**	**0.343**	**8.219**	**<0.001**
**Long cuckoo call**	**3.674**	**0.337**	**10.886**	**<0.001**
Distance to houses	0.000	0.000	0.210	0.834

**Figure 2:**
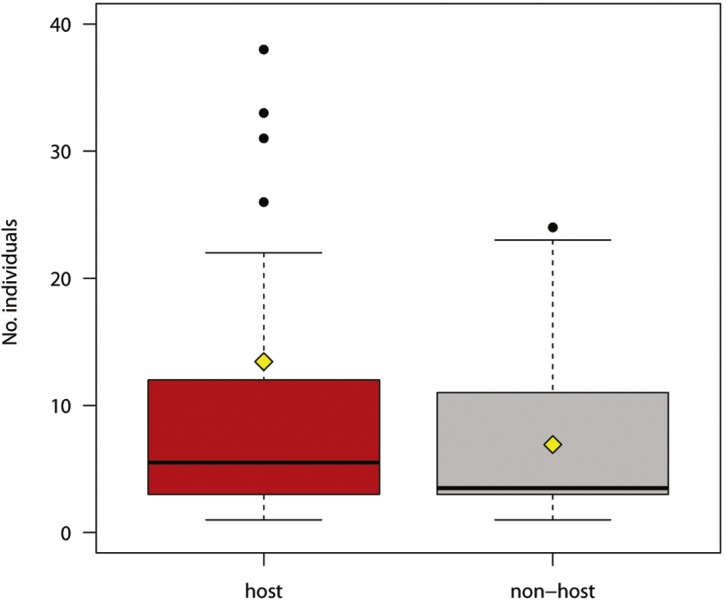
Number of individual hosts and non-hosts reacting to experimental play-back (host species in red, non-host species in grey). The boxplots show the median (black bar in the middle of rectangles), mean (yellow rhombus), upper and lower quartiles, 5- and 95-percentiles and extreme values

The number of responses was not significantly different for host and non-host species (Fig. 2; Welch two sample *t*-test for unequal variances, *t* = 1.446, *df* = 39.827, *P* = 0.156).

Birds reacted more strongly if a given species were heavily parasitized (*χ*^2^ = 7.359, *df* = 1, *P* = 0.0067, estimate (SE) = 1.053 (0.510)). Independently, colonially breeding species reacted more strongly than solitarily breeders (*χ*^2^ = 6.336, *df* = 1, *P* = 0.012, estimate (SE) = 1.053 (0.510)).

## Discussion

Cuckoos emit calls that vary in the number of repeated syllables, and such calls reflect phenotypic and habitat quality as shown by previous studies (Møller et al., 2016). Here we experimentally tested by playback of modified cuckoo calls and calls emitted by a control species, the wood pigeon, the hypothesis that duration of the call is an important factor for understanding the reaction of conspecific and host species. Birds responded differently to playback of cuckoo and woodpigeon calls differing in the number of repeat syllables, with responses being strongest to cuckoo calls with more repeats.

Cuckoo males responded much stronger to long than to short cuckoo calls, and they responded more to cuckoo than to woodpigeon control calls although woodpigeons also produce calls with repeated syllables. Thus, cuckoo males recognise potential territorial intruders, and they respond vocally to these calls as shown previously (Fuisz & De Kort, 2007; Jung, Lee & Yoo, 2014). Moreover, longer calls are not only recognised by cuckoos, but also by hosts and non-hosts. Hosts with high levels of parasitism responded more strongly in experiments and so did colonial birds. Host species with more frequent parasitism pay higher costs from the presence of a cuckoo, so selection should favour detection of the cuckoo followed by harassment (Curio, Ernst & Vieth, 1978; Davies & Welbergen, 2009). Colonial species are known for rapid detection of brood parasites and for intense harassment of cuckoos. Barn swallow *Hirundo rustica*, house martin *Delichon urbicum* and common starling *Sturnus vulgaris* have all been shown to intensely mob enemies including cuckoos, although earlier studies did not separate the effect to physical presence of cuckoos from emitted calls (Curio, Ernst & Vieth, 1978; Møller, 1987; Brown & Hoogland, 1986; Liang & Møller, 2015). Harassment by colonial birds is frequent and intense despite the fact that cuckoo parasitism of these species is exceedingly rare.

Non-host species such as corvids and thrushes also mobbed when cuckoo calls were played back, perhaps because they misinterpret visually the cuckoo as a predator such as a sparrowhawk *Accipiter nisus* (Gluckman & Mundy, 2013; Trnka & Prokop, 2012; Liang & Møller, 2015).

The response of birds to the cuckoo was stronger in experimental sites located close to human settlements, as already described (Møller, Liang & Díaz, 2016). Common cuckoo but also other bird species show a strong preference for proximity to villages in farmland areas (Rosin et al., 2016). Human habitation may be considered a refuge against raptors, because raptors keep long distances from humans and their habitation. Simultaneously, human habitation, especially cities, can be considered a refuge against the common cuckoo (Møller et al., 2016). If cuckoos compete particularly intensely for access to hosts near human habitation, where the density of hosts is the highest, and where food availability may be the highest as well, we should expect cuckoo calls near human habitation to contain more syllables. Here, we have shown that this pattern is not just correlational because play-back of cuckoo calls varying in number of syllables revealed stronger responses to cuckoo calls with more syllables, but also stronger responses to play-back of cuckoo calls near human habitation.

In conclusion, cuckoo calls function in male-male interactions, and male cuckoos producing calls with more syllables are recognised by hosts as being a higher threat of parasitism.

##  Supplemental Information

10.7717/peerj.5302/supp-1 Supplemental Information 1 Reaction of birds to presented stuffed cuckoo with different call Each line is the one experiment, arranged in following way: ID and name of the site; date 1 = 1 May, time is transformed to 0-1 value, distance to houses expressed in meters, the name of experimental trial, number of trial in the season, the reaction of birds to the cuckoo, column J, no of species reacted; K, number of individuals reacted, the other columns are species reacted to cuckoo arranged in an alphabetical list, the first 3 letters to genus and the 3 letters to the species scientific Latin name.

10.7717/peerj.5302/supp-2 Supplemental Information 2 The bird list species used in host and non-host reaction to cuckoo calls Data are arranged in columns: A, species Latin name; B, host—0—non-host, 1—host for cuckoo eggs and chocks; C, coloniality: 0-non-colonial species, 1—colonial species; D, number of interactions with cuckoo in all trials.

## Additional Information and Declarations

### Data Availability

The following information was supplied regarding data availability: The raw data are provided in the Supplemental Files.
